# Association of per- and polyfluoroalkyl substance exposure with metabolic syndrome and its components in adults and adolescents

**DOI:** 10.1007/s11356-023-30317-x

**Published:** 2023-10-16

**Authors:** Huizhen Zheng, Ziwei Yin, Xi Luo, Yingli Zhou, Fei Zhang, Zhihua Guo

**Affiliations:** 1 Department of Cardiology, The First Affiliated Hospital of Hunan University of Chinese Medicine, Changsha, 410007 China; 2 College of Chinese Medicine, Hunan University of Chinese Medicine, Changsha, 410208 China; 3 Hunan Key Laboratory of Colleges of Intelligent Traditional Chinese Medicine Diagnosis and Preventive Treatment of Chronic Diseases, Changsha, 410208 China

**Keywords:** Per- and polyfluoroalkyl substances, Metabolic syndrome, NHANES, Metabolism, Cardiovascular risk factors, Population-based study

## Abstract

Per- and polyfluoroalkyl substances (PFAS) are widespread contaminants, but few studies have explored the relationship between PFAS and levels of metabolic syndrome (MetS) in the population. The available evidence of an association is also conflicting. We selected adults and adolescents with complete PFAS data from the National Health and Nutrition Examination Survey conducted between 2003 and 2018. We analyzed the association between PFAS and MetS using multivariate logistic regression models and evaluated potential nonlinear relationships with restricted cubic spline models. Additionally, we employed weighted quantile sum (WQS) regressions to uncover the multiple exposure effects and relative weights of each PFAS. Finally, we conducted a series of sensitivity analyses to test the robustness of our findings. In this population-based study, we analyzed data from a total of 4,973 adults, aged 20–85 years, and 1,381 adolescents, aged 12–19 years. Using fully adjusted multivariate logistic regression models, we found that serum levels of perfluorodecanoate (PFDA) [0.65 (0.50, 0.85)] and total PFAS [0.92 (0.85, 0.99)] were negatively associated with the prevalence of MetS in adults. Similarly, in adolescents, we observed negative correlations between the prevalence of MetS and levels of PFDA [0.55 (0.38, 0.80)], perfluorooctanoic acid (PFOA) [0.62 (0.39, 1.00)], perfluorooctane sulfonic acid (PFOS) [0.59 (0.36, 0.96)], and total PFAS [0.61 (0.37, 0.99)]. Additionally, our study identified statistically significant negative associations between serum levels of PFAS and certain components of MetS, primarily elevated fasting glucose and lower high-density lipoprotein cholesterol. Our study found that PFAS was associated with a lower prevalence of MetS in both adults and adolescents, offering new insights into the relationship between PFAS and metabolic health. Interestingly, however, we observed conflicting findings across the components of MetS. Specifically, we observed that PFAS had a negative correlation with some metrics and a positive correlation with others. These conflicting results point to a complex interplay between PFAS and various metrics of metabolic health.

**Supplementary Information:**

The online version contains supplementary material available at 10.1007/s11356-023-30317-x.

## Introduction

Per- and polyfluoroalkyl substances (PFAS) are a class of stable, man-made chemicals widely used in industrial production and everyday products due to their resistance to oil, dirt, and water (Glüge et al. 2020). These chemicals can enter the human body through the respiratory and digestive tracts, as well as through the skin, posing a potential health risk (Zeng et al. 2022). According to the National Biomonitoring Survey, PFAS are among the most pervasive environmental contaminants today. Their long biological half-lives mean they not only persist in the environment but also accumulate in the human body (Shoeib, Harner et al. 2011). Numerous animal and human studies indicate that long-term exposure to PFAS is linked to a range of health issues, including growth retardation, tumorigenicity, hepatic and renal toxicity, immune dysfunction, endocrine disruption, and metabolic abnormalities (Feng et al. 2022). PFAS can also bind to fatty acid-binding proteins, peroxisome proliferator-activated receptors, and estrogen receptors, interfering with the normal functioning of the endocrine system (Zhang et al. 2014). This interference is thought to be one of the pathophysiological mechanisms contributing to metabolic syndrome (MetS)..

MetS is a combination of multiple risk factors such as hypertension, hyperglycemia, dyslipidemia, and abdominal obesity (Ervin 2009), and is considered an important risk factor for cardiovascular disease and diabetes. The prevalence of MetS has been on the rise in both adults and adolescents, posing a global public health concern (Li et al. 2018). As awareness about environmental pollutants like PFAS grows, an increasing number of studies are exploring their association with metabolic disorders such as dyslipidemia, hypertension, and obesity in both age groups. Adolescents with MetS risk factors are particularly vulnerable, facing diminished health and quality of life as they transition into adulthood, with elevated risks for conditions like type 2 diabetes and cardiovascular disease (Fändriks 2017). Existing research on the relationship between PFAS and MetS is inconsistent and tends to focus on adults, largely overlooking adolescents. Thus, further studies with larger sample sizes and high data quality are needed, especially in the adolescent population, to deepen our understanding of this relationship and inform more effective prevention and treatment strategies.

Many toxicological studies suggest that PFAS may influence the development of MetS (Yan et al. 2015). However, the results of some epidemiological studies have been inconsistent or conflicting. To investigate this association more thoroughly, our study utilized the latest data from the National Health and Nutrition Examination Survey (NHANES). We assessed the relationship between serum levels of PFAS and both the prevalence and components of MetS in U.S. adults and adolescents. The aim was to explore the potential mechanisms through which PFAS might influence MetS and to lay the groundwork for future research.

## Methods

### Study population

NHANES is a nationally representative cross-sectional study hosted by the National Center for Health Statistics (NCHS) to investigate the health and nutritional status of the general U.S. population (Curtin et al. 2012). This study used data collected from NHANES 2003–2018 for secondary analysis, and data were collected by physical examination, laboratory tests, questionnaires, and interviews. All participants signed written informed consent prior to participation, ensuring that the study met ethical standards and was approved by the Institutional Review Board of the Centers for Disease Control and Prevention (CDC). Detailed study design and data about NHANES are available on the official CDC website (https://www.cdc.gov/nchs/nhanes/).

**Fig. 1:**
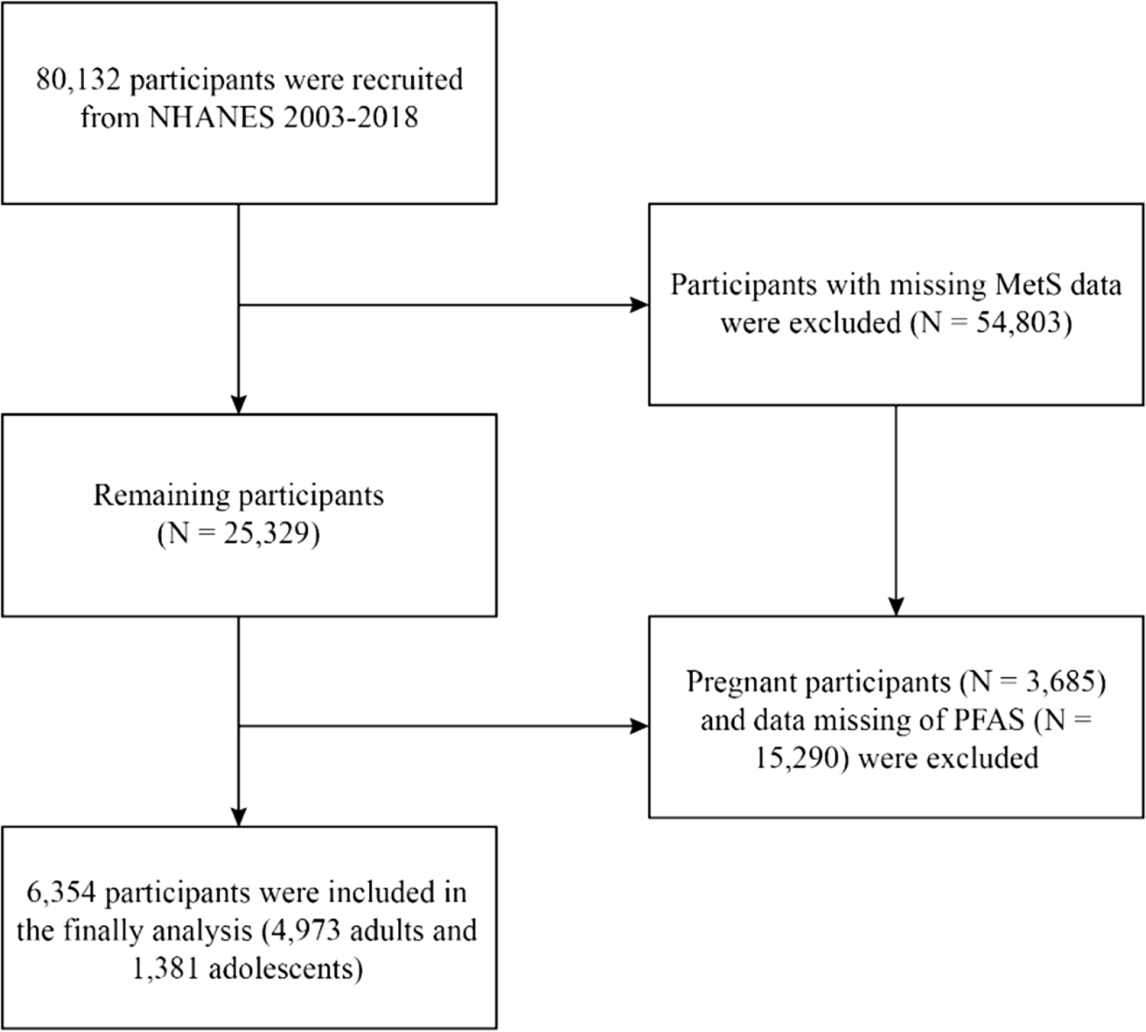
Flowchart of participant selection. Flowchart of participant selection. NHANES, National Health and Nutrition Examination Survey; PFAS, per- and polyfluoroalkyl substances; MetS, metabolic syndrome

Our study began with the recruitment of 80,132 participants from the NHANES (2003–2018) dataset. After excluding those lacking essential data such as blood pressure, high-density lipoprotein cholesterol (HDL-C), plasma fasting glucose, triglycerides (TG), and waist circumference (WC), 25,329 subjects remained. We further excluded 3,685 subjects due to pregnancy and an additional 15,290 for missing PFAS data. Ultimately, our analysis included 6,354 subjects, comprising 4,973 adults and 1,381 adolescents aged 12–19 years (Fig. 1).

### Ascertainment of MetS

To define metabolic syndrome (MetS) in adolescents, we employed a modified version of the criteria set by the National Cholesterol Education Program Adult Treatment Panel III (NCEP ATP III) (Ganji et al. 2011). The diagnostic criteria for MetS in adolescents included three or more of the following factors: (1) HDL-C ≤ 40 mg/dl (1.036 mmol/L); (2) WC ≥ 90th percentile for the same race, age, and sex; (3) TG ≥ 110 mg/dl (1.24 mmol/L); (4) fasting glucose ≥ 100 mg/dl or use of insulin or glucose-lowering medication; (5) systolic or diastolic blood pressure ≥ 90th percentile for the same race, age, and sex or on hypertensive medication.

We used the 2005 National Cholesterol Education Program (NCEP) guidelines to diagnose adult MetS (Grundy et al. 2005). According to these guidelines, an individual can be diagnosed with MetS if they meet three or more of the following criteria: (1) waist circumference: WC ≥ 102 cm in men and ≥ 88 cm in women; (2) blood pressure ≥ 130/85 mmHg or taking medication for hypertension; (3) HDL-C: HDL-C < 40 mg/dL in men and HDL-C < 50 mg/dL in women; (4) TG ≥ 150 mg/dL or use of medications; (5) fasting glucose ≥ 100 mg/L or use of insulin or glucose-lowering medications.

### Assessment of PFAS

According to the NHANES website, the investigation used online solid-phase extraction combined with high-performance liquid chromatography-turbo ionization-tandem mass spectrometry to quantify serum PFAS in participants aged 12 years and older, using a randomly selected one-third subsample with a lower limit of detection of 0.10 ng/ml. PFAS tested in the study included perfluorooctanoic acid (PFOA), perfluorohexane sulfonate (PFHxS), perfluorononanoic acid (PFNA), perfluorooctane sulfonic acid (PFOS), and perfluorodecanoate (PFDA), where total PFAS was calculated as the sum of these six substances.

### Covariates

In our study, we carefully selected potential covariates that could impact the outcomes, basing these selections on their clinical relevance. These covariates included age (years), sex (male/female), race (Mexican American/Other Hispanic/Non-Hispanic White/Non-Hispanic Black/Other race), education level (below high school/high school/above high school for adults), family income-to-poverty ratio, serum cotinine (ng/ml), energy (kcal/d), fat intake (g/d), protein intake (g/d), physical activity (< 600 MET-minutes/week and ≥ 600 MET-minutes/week). For adults, we also considered drinking (never drinkers/light drinkers/heavy drinkers), and smoking status (smokers/non-smokers).

Smokers were categorized as individuals who had smoked at least 100 cigarettes in their lifetime. Drinking status for adults was classified into three categories: never drinkers, light drinkers (≤ 10 drinks/year), and heavy drinkers (> 10 drinks/year). Physical activity was categorized according to whether the respondent met (≥ 600 MET-minutes/week, equivalent to 150 min/week of moderate-intensity or 75 min/week of vigorous-intensity physical activity) or did not meet (< 600 MET-minutes/week) the criteria recommended by the physical activity guidelines for adults. All the detailed measurement procedures for these variables are available on the official CDC website at www.cdc.gov/nchs/nhanes/.

### Statistical analysis

All statistical processes were analyzed using appropriate NHANES sampling weights and taking into account a complex multi-stage grouping survey design. The baseline characteristics table of the study population was divided into two groups based on whether the participants had MetS. Continuous variables are expressed as the mean and standard deviation (SD) or median and interquartile range (IQR), as appropriate. Categorical variables are expressed as proportions (%). Student t-test and Mann–Whitney U test were used for assessing the differences between groups for continuous variables, the former for the data of normal distribution and the latter for the data of skewed distribution. The differences of categorical variables between MetS group and non-MetS group were tested by Chi-square test. In order to adjust the skewed distributions, serum PFAS levels were natural log transformed (ln-PFAS). We examined the relationship between PFAS and MetS using multivariate logistic regression models, adjusted for all covariates. If a logistic regression analysis is conducted on a specific PFAS member in relation to MetS, the other PFAS members are also adjusted for in the analysis. We also conducted restricted cubic spline analysis to investigate the potential nonlinear dose–response relationship. We performed weighted quantile sum (WQS) regressions using the gWQS package of R. WQS is a statistical method designed to evaluate the joint association of multiple potentially correlated exposure explanatory variables on a health outcome or state, estimating the effect of a group exposure explanatory variables as a single weighted index (Carrico et al. 2015). The WQS analysis was applied for six chemical compounds. In each case, the weights are estimated by pooling the effects both positive and negative. Within the gWQS model, results were estimated by specifying the use of percentiles for exposure weighting; 40% of the data used as the test set, with the other 60% used as the validation set. We performed 1,000 bootstrapping steps in multivariate regression models and individual chemical weights of > 0.14 were considered more important (Carrico et al. 2015). Additionally, we conducted a series of sensitivity analyses to assess the robustness of our results. For adults, we incorporated gender as a categorical variable in a fully adjusted model, termed 'sensitivity i'. In subsequent reanalyses, age was categorized as young (20–39 years old), middle-aged (40–59 years old), and old (≥ 60 years old) (sensitivity ii). We also added smoking status and race as categorical variables, termed 'sensitivity iii' and 'sensitivity iv,' respectively. For the adolescent sensitivity analyses, gender was included as a categorical variable in the fully adjusted model ('sensitivity i'), followed by the inclusion of race as a categorical variable ('sensitivity ii'). All statistical analyses were performed using R (version 4.2.1) and EmpowerStats (version: 4.1). P < 0.05 was considered statistically significant.

## Results

### Baseline characteristics

Tables 1 and 2 present the baseline characteristics of adults and adolescents, respectively. The mean exposure levels of PFAS for all participants were as follows: PFDA at 0.34 ± 0.53ng/ml, PFHxS at 2.26 ± 2.70ng/ml, PFNA at 1.16 ± 1.47ng/ml, PFOA at 3.31 ± 2.98ng/ml, PFOS at 13.37 ± 15.63ng/ml, and total PFAS at 20.44 ± 19.75ng/ml. In adults, the levels were PFOS at 13.23 ± 15.13ng/ml, PFOA at 3.47 ± 3.12ng/ml, PFDA at 0.34 ± 0.48ng/ml, PFHxS at 2.19 ± 2.34ng/ml, PFNA at 1.25 ± 1.51ng/ml, and total PFAS at 20.48 ± 19.24ng/ml. For adolescents, the levels were PFOS at 9.88 ± 9.17ng/ml, PFOA at 2.93 ± 1.97ng/ml, PFDA at 0.24 ± 0.20ng/ml, PFHxS at 2.84 ± 4.01ng/ml, PFNA at 0.97 ± 0.74ng/ml, and total PFAS at 16.86 ± 13.78ng/ml. The prevalence of MetS in adults and adolescents was 40.04% and 9.49%, respectively. Among the 4,973 adults, the mean age of participants was 47.74 ± 16.88 years (range 20–85 years) and included 2,497 (50.21%) males and 2,476 (49.79%) females, of whom 7.88% were Mexican American, 69.53% were non-Hispanic white, 10.75% were non-Hispanic black, 4.92% were other Hispanic, and 6.92% were from other races. Of the 1,381 adolescents, the mean age of participants was 15.55 ± 2.28 years (range 12–19 years), including 749 (54.24%) males and 632 (45.76%) females, of whom 14.68% were Mexican American, 56.64% were non-Hispanic White, 14.78% were non-Hispanic Black, 6.41% were other Hispanic, and 7.49% were from other races.

**Table 1:** Weighted comparison in basic characteristics in adult participants

	Overall (*n* = 4,973)	MetS (*n* = 1,991)	Non-MetS (*n* = 2,982)	*P*-value
Gender (%)				< 0.001^c^
Male	50.41	54.82	47.84	
Female	49.59	45.18	52.16	
Age (mean (SD))	47.74 ± 16.88	44.22 ± 16.72	53.78 ± 15.39	< 0.0001^a^
Race/Ethnicity (%)				0.0015^c^
Mexican American	7.88	8.55	7.49	
Other Hispanic	4.92	4.6	5.11	
Non-Hispanic white	69.53	71.72	68.25	
Non-Hispanic black	10.75	9.84	11.29	
Other race or multi-racial	6.92	5.3	7.86	
Education level (%)				< 0.0001^c^
Less than high school	15.99	20.01	13.65	
High school	24.41	27.21	22.78	
More than high school	59.6	52.78	63.56	
Smoked at least 100 cigarettes (%)				< 0.001^c^
Yes	46.37	50.4	44.02	
No	53.63	49.6	55.98	
Alcohol use (%)				0.955^c^
Never drinkers	24.9	25.09	24.78	
Light drinkers	71.39	71.26	71.47	
Heavy drinkers	3.71	3.65	3.75	
Physical activity (%)				< 0.0001^c^
< 600 MET-minutes/week	61.03	67.61	50.59	
≥ 600 MET-minutes/week	38.97	32.39	49.41	
Income to poverty ratio	2.65 ± 1.66	2.70 ± 1.60	2.65 ± 1.61	0.5201^a^
Energy Intake (kcal/d)	2125.69 ± 818.60	2118.25 ± 823.55	2130.14 ± 815.60	0.6461^a^
Protein Intake (g/d)	83.43 ± 34.79	83.60 ± 35.06	83.33 ± 34.62	0.8065^a^
Fat intake (g/d)	82.28 ± 38.28	83.71 ± 39.39	81.42 ± 37.57	0.0587^a^
PFOS (median [IQR])*	13.23 ± 15.13	14.32 ± 16.33	12.60 ± 14.35	0.0001^b^
PFOA (median [IQR])*	3.47 ± 3.12	3.49 ± 2.72	3.45 ± 3.32	0.6941^b^
PFDA (median [IQR])*	0.34 ± 0.48	0.32 ± 0.35	0.36 ± 0.54	0.0073^b^
PFHxS (median [IQR])*	2.19 ± 2.34	2.25 ± 2.48	2.16 ± 2.25	0.1777^b^
PFNA (median [IQR])*	1.25 ± 1.51	1.24 ± 1.05	1.25 ± 1.72	0.7106^b^
Total PFAS (median [IQR])*	20.48 ± 19.24	21.62 ± 20.29	19.82 ± 18.57	0.0015^b^
Serum cotinine (ng/ml)	60.74 ± 131.10	59.73 ± 132.14	61.33 ± 130.49	0.6773^a^

a: Student's t-test was used to compare normally distributed continuous variables between MetS and non-MetS groups

b: Mann–Whitney U test was used to test the distributions of skewed variables between MetS and non-MetS groups

c: Chi-square test was used to test the distributions of categorical variables between MetS and non-MetS groups

PFAS, per- and polyfluoroalkyl substances; PFDA, perfluorodecanoate; PFHxS, perfluorohexane sulfonate; PFNA, per fluorononanoic acid; PFOA, perfluorooctanoic acid; PFOS, perfluorooctane sulfonic acid; MetS, metabolic syndrome; MET, metabolic equivalent of task; ⁎unit: ng/ml

**Table 2:** Weighted comparison in basic characteristics in adolescent participants

	Overall (*n* = 1,381)	MetS (*n* = 131)	Non-MetS (*n* = 1,250)	*P*-value
Gender (%)				0.0002^c^
Male	54.35	69.74	52.74	
Female	45.65	30.26	47.26	
Age (mean (SD))	15.55 ± 2.28	15.94 ± 2.38	15.51 ± 2.26	0.0406^a^
Race/Ethnicity (%)				0.0049^c^
Mexican American	14.68	23.22	13.78	
Other Hispanic	6.41	8.71	6.17	
Non-Hispanic white	56.64	53.21	57	
Non-Hispanic black	14.78	12.87	14.98	
Other race or multi-racial	7.49	1.99	8.07	
Income to poverty ratio	2.48 ± 1.62	2.19 ± 1.39	2.51 ± 1.64	0.0289^a^
Energy Intake (kcal/d)	76.76 ± 33.17	77.53 ± 35.33	76.68 ± 32.93	0.7905^a^
Protein Intake (g/d)	78.60 ± 37.19	78.29 ± 41.23	78.63 ± 36.73	0.9248^a^
Fat intake (g/d)	19.09 ± 68.80	19.44 ± 74.47	19.05 ± 68.18	0.9509^a^
PFOS (median [IQR])*	9.88 ± 9.17	8.50 ± 7.94	10.03 ± 9.27	0.0693^b^
PFOA (median [IQR])*	2.93 ± 1.97	2.67 ± 1.71	2.96 ± 1.99	0.106^b^
PFDA (median [IQR])*	0.24 ± 0.20	0.21 ± 0.15	0.24 ± 0.21	0.1146^b^
PFHxS (median [IQR])*	2.84 ± 4.01	2.07 ± 2.50	2.92 ± 4.13	0.0216^b^
PFNA (median [IQR])*	0.97 ± 0.74	0.98 ± 0.74	0.97 ± 0.74	0.8233^b^
Total PFAS (median [IQR])*	16.86 ± 13.78	14.43 ± 11.08	17.11 ± 14.01	0.0341^b^
Serum cotinine (ng/ml)	19.09 ± 68.80	19.44 ± 74.47	19.05 ± 68.18	0.9509^a^

a: Student's t-test was used to compare normally distributed continuous variables between MetS and non-MetS groups

b: Mann–Whitney U test was used to test the distributions of skewed variables between MetS and non-MetS groups

c: Chi-square test was used to test the distributions of categorical variables between MetS and non-MetS groups

PFAS, per- and polyfluoroalkyl substances; PFDA, perfluorodecanoate; PFHxS, perfluorohexane sulfonate; PFNA, per fluorononanoic acid; PFOA, perfluorooctanoic acid; PFOS, perfluorooctane sulfonic acid; MetS, metabolic syndrome; ⁎unit: ng/ml

We observed significant differences in both demographic and baseline clinical characteristics between MetS patients and non-MetS patients. Among adults, relative to the non-MetS group, MetS participants were more likely to be male, middle-aged, Mexican American and non-Hispanic white, smokers, with lower levels of education and lower time for physical activity. For chemical exposure, except for PFDA, levels of PFOS and total PFAS were higher in the MetS group; PFOA, PFHxS, and PFNA differences did not reach statistical significance (*P* > 0.05). In addition, among adolescents, MetS participants were more likely to be male, Mexican American and other Hispanic compared to the non-MetS group. Notably, PFHxS and total PFAS levels were lower in the MetS group compared to the non-MetS group, while PFOA, PFOS, PFDA, and PFNA differences were not statistically significant (*P* > 0.05). In both adults and adolescents, MetS subjects were more likely to be male Mexican Americans compared to non-MetS subjects.

### Association between PFAS and MetS

Tables 3 and 4 shows the association between serum PFAS levels and the prevalence of MetS in both adults and adolescents. For adults, after adjusting for all covariates, we found that each unit increase in the log-transformed levels of PFDA [0.65 (0.50, 0.85)] and total PFAS [0.92 (0.85, 0.99)] was significantly linked to a reduction in MetS prevalence. Specifically, lower concentrations of PFDA [0.82 (0.70, 0.96)], PFNA [0.77 (0.66, 0.90)], and PFOA [0.79 (0.68, 0.93)], as well as higher concentrations of total PFAS [0.84 (0.72, 0.99)] and PFHxS [0.85 (0.73, 1.00)], were associated with reduced MetS prevalence. However, the link between PFOS [0.93 (0.87, 1.00)] and MetS did not reach statistical significance. In adolescents, we observed a reduction in MetS prevalence with each 1-unit increase in log-transformed levels, but this was only statistically significant for higher concentrations of PFDA [0.55 (0.38, 0.80)], PFOA [0.62 (0.39, 1.00)], PFOS [0.59 (0.36, 0.96)], and total PFAS [0.61 (0.37, 0.99)].

**Table 3:** Odds ratios (ORs) and 95% confidence intervals (CIs) for association between serum PFAS levels and metabolic syndrome in adult participants

	PFDA	PFHxS	PFNA	PFOA	PFOS	Total PFAS
Continuous (Ln-transformed OR (95%CI) P value)						
	0.65 (0.50, 0.85) 0.0019	0.91 (0.81, 1.03) 0.1258	0.90 (0.76, 1.06) 0.1993	0.92 (0.82, 1.04) 0.1660	0.93 (0.87, 1.00) 0.0650	0.92 (0.85, 0.99) 0.0346
Categories						
Tertile 1	Reference	Reference	Reference	Reference	Reference	Reference
Tertile 2	0.82 (0.70, 0.96) 0.0153	0.86 (0.74, 1.01) 0.0602	0.77 (0.66, 0.90) 0.0007	0.79 (0.68, 0.93) 0.0031	0.90 (0.77, 1.04) 0.1550	0.89 (0.77, 1.04) 0.1477
Tertile 3	0.87 (0.75, 1.00) 0.0543	0.85 (0.73, 1.00) 0.0485	0.93 (0.80, 1.09) 0.3670	0.94 (0.81, 1.10) 0.4476	0.87 (0.75, 1.02) 0.0898	0.84 (0.72, 0.99) 0.0339
*P* for trend	0.1493	0.0645	0.6262	0.5957	0.0886	0.0332

PFAS, per- and polyfluoroalkyl substances; PFDA, perfluorodecanoate; PFHxS, perfluorohexane sulfonate; PFNA, perfluorononanoic acid; PFOA, perfluorooctanoic acid; PFOS, perfluorooctane sulfonic acid

**Table 4:** Odds ratios (ORs) and 95% confidence intervals (CIs) for association between serum PFAS levels and metabolic syndrome in adolescent participants

	PFDA	PFHxS	PFNA	PFOA	PFOS	Total PFAS
Continuous (Ln-transformed OR (95%CI) P value)						
	0.23 (0.05, 1.16) 0.0752	0.79 (0.57, 1.09) 0.1482	1.01 (0.55, 1.87) 0.9735	0.75 (0.50, 1.13) 0.1710	0.82 (0.65, 1.04) 0.1000	0.81 (0.62, 1.05) 0.1100
Categories						
Tertile 1	Reference	Reference	Reference	Reference	Reference	Reference
Tertile 2	0.32 (0.04, 2.47) 0.2754	0.83 (0.53, 1.32) 0.4340	1.00 (0.62, 1.61) 0.9993	0.88 (0.56, 1.37) 0.5639	0.98 (0.64, 1.52) 0.9335	0.94 (0.61, 1.46) 0.7789
Tertile 3	0.55 (0.38, 0.80) 0.0019	0.70 (0.43, 1.13) 0.1467	1.07 (0.67, 1.70) 0.7908	0.62 (0.39, 1.00) 0.0484	0.59 (0.36, 0.96) 0.0334	0.61 (0.37, 0.99) 0.0435
*P* for trend	0.0017	0.1578	0.7706	0.0524	0.0515	0.0581

PFAS, per- and polyfluoroalkyl substances; PFDA, perfluorodecanoate; PFHxS, perfluorohexane sulfonate; PFNA, perfluorononanoic acid; PFOA, perfluorooctanoic acid; PFOS, perfluorooctane sulfonic acid

The relationship between serum PFAS and MetS components differed between adults and adolescents, as outlined in Tables 5 and 6. In adults, after adjusting for all relevant variables, each unit increase in the log-transformed levels of PFNA [0.75 (0.63, 0.88)], PFHxS [0.87 (0.77, 0.99)], PFOA [0.80 (0.71, 0.91)], PFOS [0.84 (0.78, 0.90)], and total PFAS [0.82 (0.76, 0.89)] corresponded to a one-unit reduction in the risk of elevated fasting glucose (EGLU). Further, each one-unit increase in serum log-transformed levels of each PFAS was linked with a one-unit risk reduction in reduced HDL-C. The specific odds ratios (OR) and 95% confidence intervals for these substances were as follows: PFDA at 0.48 (0.34, 0.66), PFHxS at 0.75 (0.66, 0.86), PFNA at 0.76 (0.64, 0.92), PFOA at 0.81 (0.71, 0.91), PFOS at 0.87 (0.80, 0.94), and total PFAS at 0.82 (0.76, 0.90). For adolescents, the results were somewhat different. PFHxS [0.75 (0.57, 0.98)], PFOS [0.80 (0.66, 0.97)], and total PFAS [0.99 (0.97, 1.00)] were similarly associated with a decreased risk of reduced HDL-C. Unexpectedly, PFDA [3.03 (1.32, 6.96)] was associated with an increased risk of a larger waist circumference. Furthermore, when adolescent serum PFAS levels were categorized using triple-cohortization, low concentrations of PFDA [0.60 (0.40, 0.91)] was also associated with an elevated risk of elevated triglycerides.

**Table 5:** Odds ratios (ORs) and 95% confidence intervals (CIs) for association between serum PFAS levels and metabolic syndrome components in adult participants

	Elevated blood pressure	Elevated glucose	Reduced HDL cholesterol	Elevated triglycerides	Increased waist circumference
Continuous (Ln-transformed OR (95%CI) P value)					
PFDA	0.79 (0.59, 1.06) 0.1137	0.83 (0.64, 1.08) 0.1641	0.48 (0.34, 0.66) < 0.0001	0.99 (0.75, 1.31) 0.9492	1.01 (0.78, 1.31) 0.9385
PFHxS	1.02 (0.89, 1.17) 0.7537	0.87 (0.77, 0.99) 0.0279	0.75 (0.66, 0.86) < 0.0001	0.98 (0.86, 1.12) 0.7856	1.02 (0.90, 1.15) 0.7685
PFNA	0.86 (0.72, 1.03) 0.1076	0.75 (0.63, 0.88) 0.0005	0.76 (0.64, 0.92) 0.0041	0.96 (0.80, 1.14) 0.6123	1.05 (0.89, 1.23) 0.5913
PFOA	0.91 (0.80, 1.03) 0.1384	0.80 (0.71, 0.91) 0.0003	0.81 (0.71, 0.91) 0.0008	0.96 (0.85, 1.09) 0.5406	1.03 (0.91, 1.15) 0.6586
PFOS	0.94 (0.87, 1.02) 0.1219	0.84 (0.78, 0.90) < 0.0001	0.87 (0.80, 0.94) 0.0005	0.97 (0.89, 1.04) 0.3896	1.00 (0.93, 1.07) 0.9357
Total PFAS	0.93 (0.85, 1.02) 0.1101	0.82 (0.76, 0.89) < 0.0001	0.82 (0.76, 0.90) < 0.0001	0.96 (0.88, 1.05) 0.3777	1.00 (0.93, 1.08) 0.9680
Categories					
PFDA					
Tertile 1	Reference	Reference	Reference	Reference	Reference
Tertile 2	0.93 (0.78, 1.12) 0.4497	0.78 (0.66, 0.92) 0.0027	0.70 (0.59, 0.83) < 0.0001	1.02 (0.86, 1.22) 0.8100	0.97 (0.83, 1.14) 0.7485
Tertile 3	0.87 (0.74, 1.02) 0.0877	0.97 (0.84, 1.12) 0.6957	0.71 (0.61, 0.83) < 0.0001	1.00 (0.86, 1.17) 0.9707	1.04 (0.90, 1.20) 0.5889
*P* for trend	0.0885	0.6552	0.0001	0.9743	0.4785
PFHxS					
Tertile 1	Reference	Reference	Reference	Reference	Reference
Tertile 2	0.93 (0.78, 1.09) 0.3618	0.91 (0.78, 1.06) 0.2167	0.76 (0.65, 0.89) 0.0007	1.04 (0.88, 1.23) 0.6236	1.09 (0.94, 1.27) 0.2714
Tertile 3	0.98 (0.82, 1.16) 0.7775	0.85 (0.72, 0.99) 0.0424	0.69 (0.58, 0.82) < 0.0001	0.91 (0.77, 1.08) 0.2956	1.02 (0.87, 1.19) 0.7924
*P* for trend	0.8498	0.0447	< 0.0001	0.2273	0.9057
PFNA					
Tertile 1	Reference	Reference	Reference	Reference	Reference
Tertile 2	0.92 (0.78, 1.09) 0.3467	0.76 (0.66, 0.89) 0.0005	0.78 (0.66, 0.91) 0.0017	1.03 (0.88, 1.21) 0.7356	0.92 (0.79, 1.06) 0.2459
Tertile 3	0.94 (0.79, 1.11) 0.4433	0.74 (0.64, 0.87) 0.0002	0.82 (0.69, 0.96) 0.0155	0.93 (0.79, 1.10) 0.4036	0.99 (0.85, 1.15) 0.8914
*P* for trend	0.4858	0.0005	0.0249	0.3472	0.9890
PFOA					
Tertile 1	Reference	Reference	Reference	Reference	Reference
Tertile 2	0.96 (0.81, 1.13) 0.5872	0.78 (0.67, 0.91) 0.0013	0.78 (0.67, 0.91) 0.0022	1.04 (0.89, 1.23) 0.6064	0.94 (0.81, 1.10) 0.4454
Tertile 3	0.94 (0.80, 1.12) 0.4884	0.75 (0.64, 0.87) 0.0002	0.82 (0.70, 0.97) 0.0190	0.92 (0.78, 1.09) 0.3589	0.97 (0.84, 1.13) 0.7427
*P* for trend	0.4968	0.0003	0.0232	0.3156	0.7817
PFOS					
Tertile 1	Reference	Reference	Reference	Reference	Reference
Tertile 2	0.96 (0.82, 1.13) 0.6412	0.90 (0.77, 1.04) 0.1489	0.82 (0.70, 0.96) 0.0113	1.09 (0.93, 1.28) 0.3006	1.00 (0.86, 1.16) 0.9922
Tertile 3	0.89 (0.75, 1.05) 0.1745	0.71 (0.61, 0.83) < 0.0001	0.76 (0.65, 0.90) 0.0016	0.93 (0.78, 1.10) 0.4063	1.00 (0.85, 1.16) 0.9552
*P* for trend	0.1770	< 0.0001	0.0013	0.4130	0.9555
Total PFAS					
Tertile 1	Reference	Reference	Reference	Reference	Reference
Tertile 2	0.96 (0.82, 1.13) 0.6226	0.85 (0.73, 0.99) 0.0367	0.81 (0.69, 0.94) 0.0072	0.99 (0.84, 1.16) 0.8725	0.98 (0.84, 1.13) 0.7557
Tertile 3	0.93 (0.79, 1.11) 0.4391	0.70 (0.60, 0.82) < 0.0001	0.73 (0.62, 0.87) 0.0003	0.88 (0.74, 1.04) 0.1382	1.00 (0.85, 1.16) 0.9748
*P* for trend	0.4369	< 0.0001	0.0002	0.1426	0.9686

PFAS, per- and polyfluoroalkyl substances; PFDA, perfluorodecanoate; PFHxS, perfluorohexane sulfonate; PFNA, perfluorononanoic acid; PFOA, perfluorooctanoic acid; PFOS, perfluorooctane sulfonic acid

**Table 6:** Odds ratios (ORs) and 95% confidence intervals (CIs) for association between serum PFAS levels and metabolic syndrome components in adolescent participants

	Elevated blood pressure	Elevated glucose	Reduced HDL cholesterol	Elevated triglycerides	Increased waist circumference
Continuous (Ln-transformed OR (95%CI) P value)					
PFDA	0.27 (0.04, 1.67) 0.1598	1.73 (0.74, 4.08) 0.2081	0.32 (0.09, 1.14) 0.0795	1.28 (0.51, 3.17) 0.5990	3.03 (1.32, 6.96) 0.0089
PFHxS	0.72 (0.50, 1.04) 0.0798	0.94 (0.76, 1.17) 0.5959	0.75 (0.57, 0.98) 0.0332	0.94 (0.75, 1.17) 0.5738	0.95 (0.77, 1.16) 0.5971
PFNA	0.62 (0.30, 1.28) 0.1979	1.02 (0.66, 1.58) 0.9378	0.94 (0.56, 1.57) 0.8043	0.82 (0.52, 1.29) 0.3890	1.18 (0.78, 1.81) 0.4341
PFOA	0.80 (0.50, 1.26) 0.3276	1.23 (0.92, 1.64) 0.1636	0.75 (0.53, 1.05) 0.0911	0.83 (0.62, 1.12) 0.2193	1.01 (0.76, 1.33) 0.9610
PFOS	0.84 (0.64, 1.10) 0.1998	1.14 (0.96, 1.35) 0.1232	0.80 (0.66, 0.97) 0.0232	0.94 (0.79, 1.11) 0.4746	1.02 (0.87, 1.20) 0.7985
Total PFAS	0.99 (0.97, 1.00) 0.0852	1.00 (0.99, 1.01) 0.4323	0.99 (0.97, 1.00) 0.0282	1.00 (0.99, 1.01) 0.9643	1.00 (0.99, 1.01) 0.6742
Categories					
PFDA					
Tertile 1	Reference	Reference	Reference	Reference	Reference
Tertile 2	1.01 (0.22, 4.69) 0.9884	0.74 (0.27, 2.03) 0.5572	0.72 (0.21, 2.50) 0.6029	2.96 (1.31, 6.69) 0.0091	0.91 (0.34, 2.38) 0.8408
Tertile 3	0.76 (0.48, 1.20) 0.2390	0.91 (0.69, 1.20) 0.4976	0.80 (0.58, 1.10) 0.1739	1.02 (0.76, 1.37) 0.8838	1.31 (1.00, 1.72) 0.0535
*P* for trend	0.2427	0.4872	0.1705	0.8097	0.0562
PFHxS					
Tertile 1	Reference	Reference	Reference	Reference	Reference
Tertile 2	0.80 (0.47, 1.36) 0.4121	0.79 (0.57, 1.10) 0.1621	0.90 (0.62, 1.30) 0.5673	1.01 (0.72, 1.41) 0.9506	0.87 (0.64, 1.18) 0.3571
Tertile 3	0.57 (0.32, 1.00) 0.0502	0.87 (0.62, 1.21) 0.3990	0.60 (0.40, 0.91) 0.0149	0.96 (0.68, 1.36) 0.8227	0.79 (0.57, 1.08) 0.1415
*P* for trend	0.0485	0.5784	0.0104	0.7875	0.1602
PFNA					
Tertile 1	Reference	Reference	Reference	Reference	Reference
Tertile 2	1.06 (0.61, 1.84) 0.8446	1.17 (0.83, 1.63) 0.3716	1.14 (0.77, 1.68) 0.5132	0.66 (0.47, 0.93) 0.0179	0.83 (0.60, 1.14) 0.2474
Tertile 3	0.75 (0.43, 1.33) 0.3272	1.10 (0.78, 1.55) 0.5818	0.92 (0.62, 1.37) 0.6781	0.78 (0.56, 1.08) 0.1323	1.02 (0.75, 1.40) 0.8878
*P* for trend	0.2414	0.6866	0.5487	0.2352	0.7048
PFOA					
Tertile 1	Reference	Reference	Reference	Reference	Reference
Tertile 2	0.89 (0.51, 1.54) 0.6705	1.07 (0.77, 1.50) 0.6835	0.70 (0.47, 1.02) 0.0636	0.92 (0.66, 1.28) 0.6298	1.02 (0.75, 1.38) 0.9121
Tertile 3	0.77 (0.46, 1.31) 0.3430	1.28 (0.92, 1.79) 0.1373	0.69 (0.48, 1.01) 0.0583	0.84 (0.60, 1.17) 0.3081	1.05 (0.77, 1.44) 0.7545
*P* for trend	0.3430	0.1404	0.0503	0.3097	0.7580
PFOS					
Tertile 1	Reference	Reference	Reference	Reference	Reference
Tertile 2	0.95 (0.55, 1.64) 0.8650	1.13 (0.81, 1.58) 0.4651	0.93 (0.64, 1.35) 0.7042	0.89 (0.64, 1.23) 0.4814	0.97 (0.71, 1.32) 0.8262
Tertile 3	0.82 (0.48, 1.41) 0.4738	1.22 (0.87, 1.70) 0.2469	0.62 (0.42, 0.93) 0.0193	0.84 (0.59, 1.17) 0.2994	1.11 (0.81, 1.53) 0.5088
*P* for trend	0.4804	0.2446	0.0260	0.2909	0.5512
Total PFAS					
Tertile 1	Reference	Reference	Reference	Reference	Reference
Tertile 2	1.15 (0.67, 1.99) 0.6071	1.15 (0.83, 1.60) 0.4082	1.05 (0.73, 1.51) 0.8046	0.93 (0.67, 1.30) 0.6840	0.93 (0.68, 1.27) 0.6457
Tertile 3	0.85 (0.49, 1.47) 0.5657	1.26 (0.90, 1.75) 0.1820	0.60 (0.40, 0.90) 0.0138	0.83 (0.59, 1.17) 0.2976	1.03 (0.75, 1.42) 0.8480
*P* for trend	0.4607	0.1949	0.0091	0.2948	0.7928

PFAS, per- and polyfluoroalkyl substances; PFDA, perfluorodecanoate; PFHxS, perfluorohexane sulfonate; PFNA, perfluorononanoic acid; PFOA, perfluorooctanoic acid; PFOS, perfluorooctane sulfonic acid

**Fig. 2:**
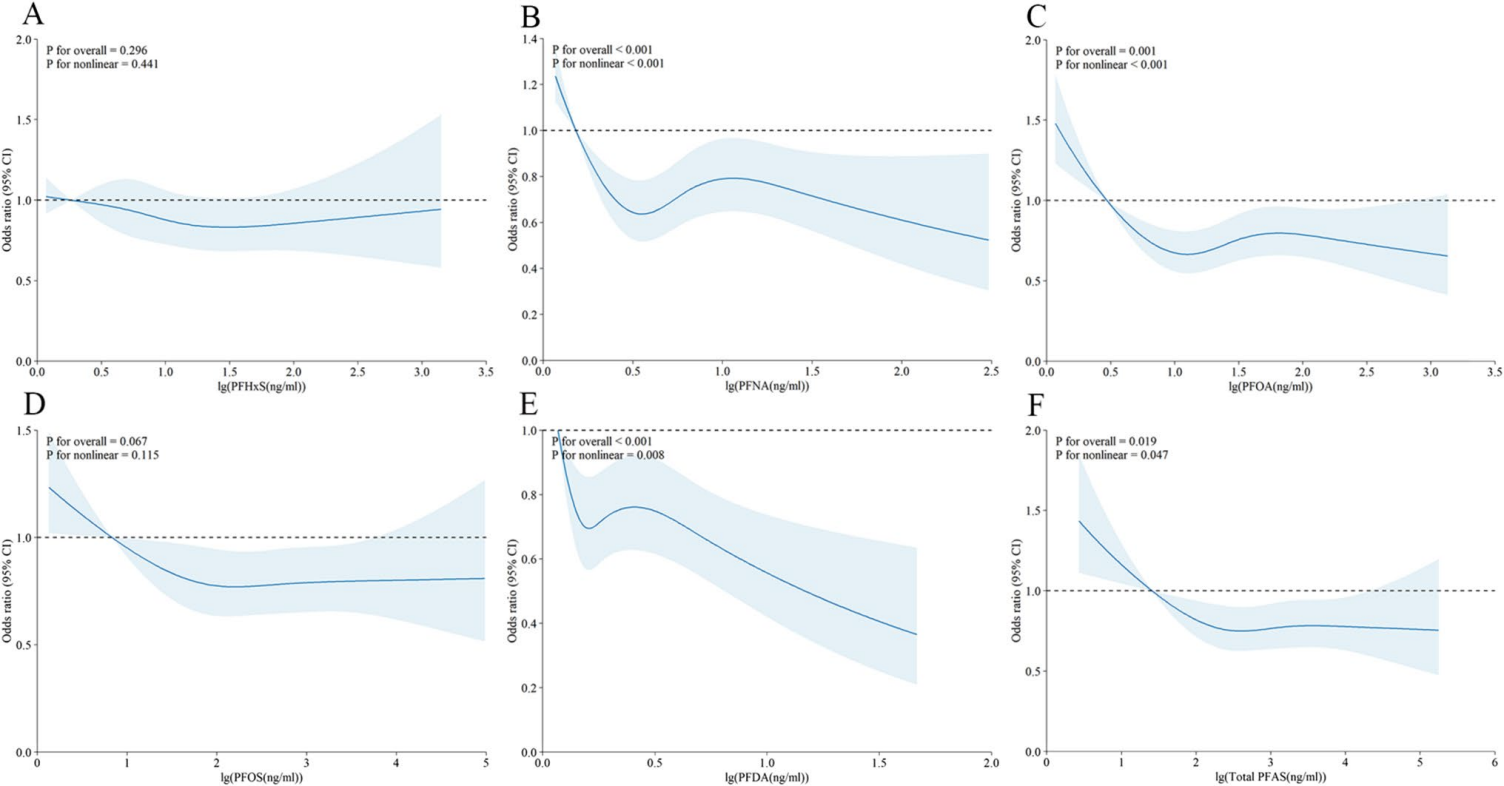
Restricted cubic spline of the association between serum PFAS concentration and MetS in adult participants. **A**. PFHxS; **B**. PFNA; **C**. PFOA; **D**. PFOS; **E**. PFDA; **F**. total PFAS. PFAS, per- and polyfluoroalkyl substances; PFDA, perfluorodecanoate; PFHxS, perfluorohexane sulfonate; PFNA, perfluorononanoic acid; PFOA, perfluorooctanoic acid; PFOS, perfluorooctane sulfonic acid. MetS, metabolic syndrome. The solid line represents the estimated RR, and the shaded part represents the 95% confidence interval

**Fig. 3:**
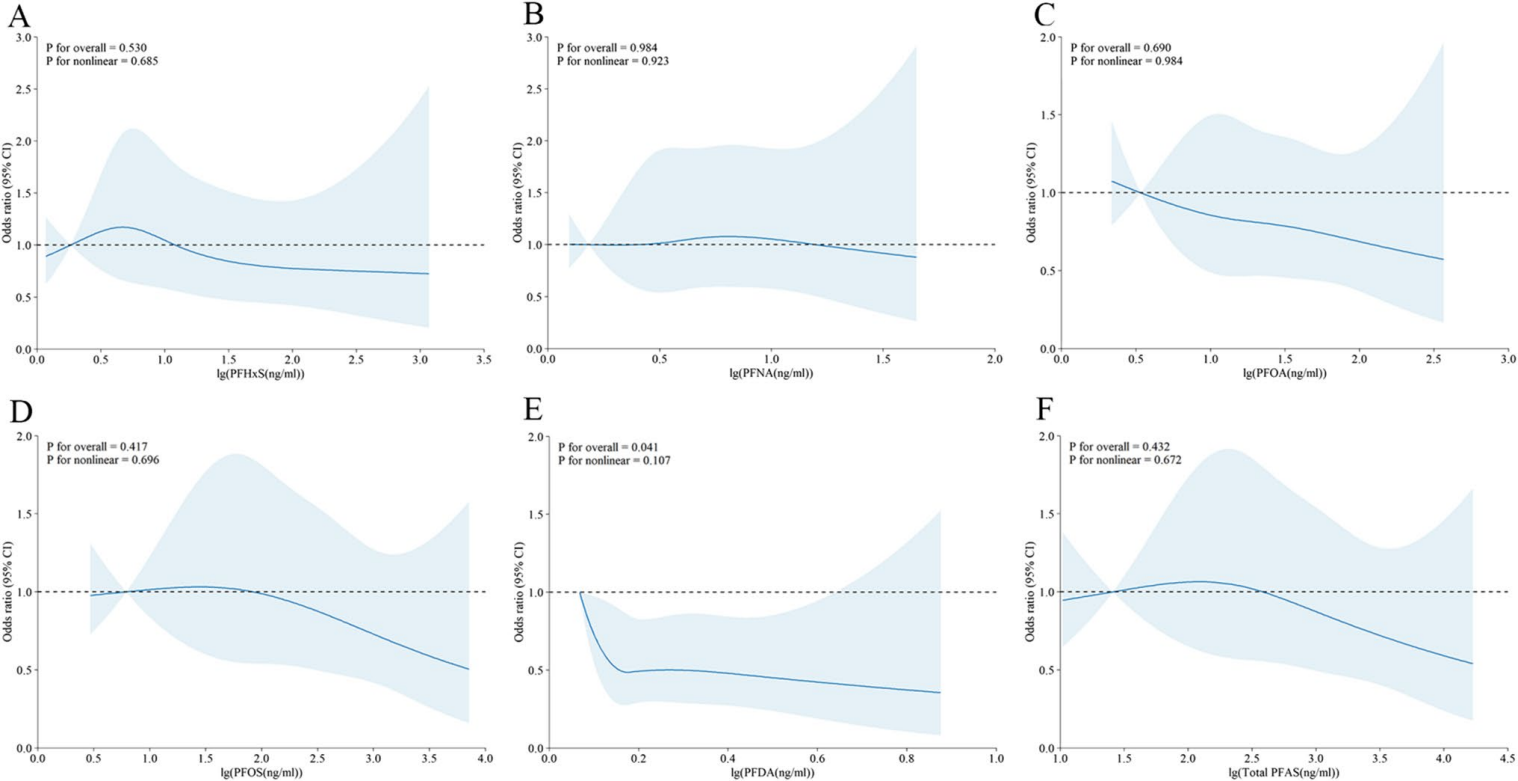
Restricted cubic spline of the association between serum PFAS concentration and MetS in adolescent participants. **A**. PFHxS; **B**. PFNA; **C**. PFOA; **D**. PFOS; **E**. PFDA; **F**. total PFAS. PFAS, per- and polyfluoroalkyl substances; PFDA, perfluorodecanoate; PFHxS, perfluorohexane sulfonate; PFNA, perfluorononanoic acid; PFOA, perfluorooctanoic acid; PFOS, perfluorooctane sulfonic acid. MetS, metabolic syndrome. The solid line represents the estimated RR, and the shaded part represents the 95% confidence interval

We examined the potential for nonlinear dose–response relationships, and the results are presented in Figs. 2 and 3. In adults (as shown in Fig. 2), we found nonlinear associations between MetS risk and the following PFAS compounds: PFNA (p for nonlinearity < 0.001), PFOA (p for nonlinearity = 0.001), PFDA (p for nonlinearity < 0.001), and total PFAS (p for nonlinearity = 0.019). However, we did not detect any nonlinear associations between MetS risk and either PFHxS (p for nonlinearity = 0.296) or PFOS (p for nonlinearity = 0.067). In adolescents (as shown in Fig. 3), a nonlinear relationship was observed only between PFDA and MetS risk (p = 0.041). No nonlinear associations were found between MetS risk and PFHxS (p for nonlinearity = 0.530), PFNA (p for nonlinearity = 0.984), PFOA (p for nonlinearity = 0.690), PFOS (p for nonlinearity = 0.417), or total PFAS (p for nonlinearity = 0.432).

### Weight analysis

**Fig. 4:**
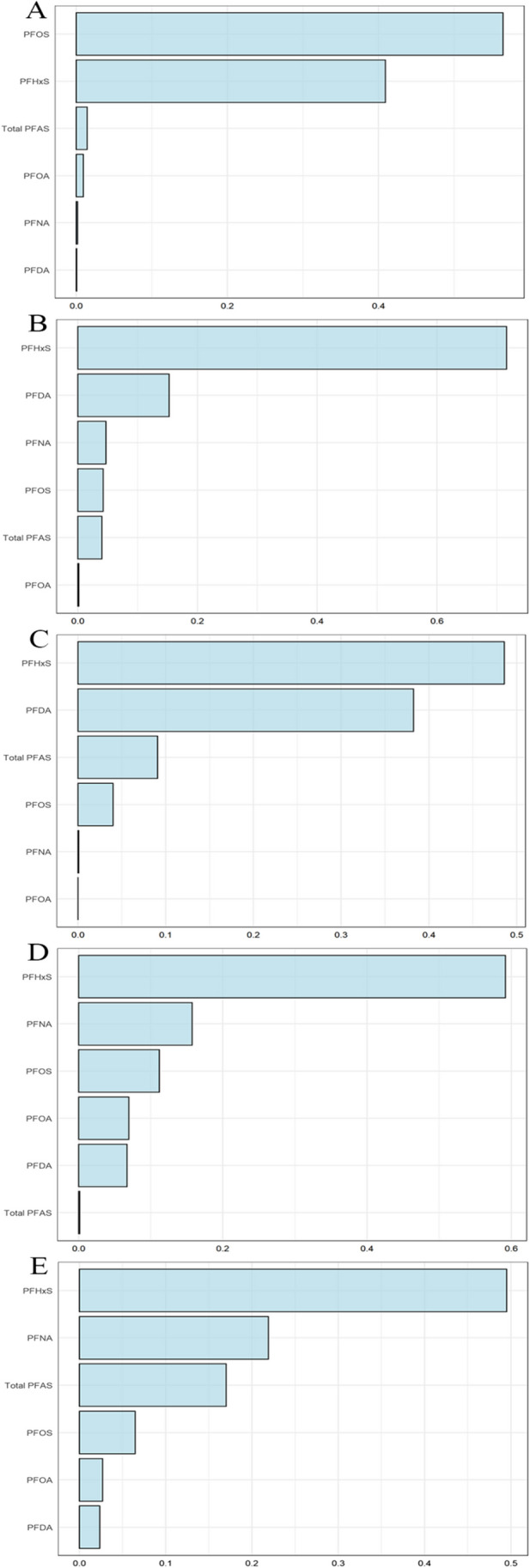
Contribution of PFAS to WQS percentage for metabolic syndrome in adult participants. **A**. The WQS index weights for elevated blood pressure. **B**. The WQS index weights for elevated glucose. **C**. The WQS index weights for reduced HDL cholesterol. **D**. The WQS index weights for elevated triglycerides. **E**. The WQS index weights for increased waist circumference. PFAS, per- and polyfluoroalkyl substances; PFDA, perfluorodecanoate; PFHxS, perfluorohexane sulfonate; PFNA, perfluorononanoic acid; PFOA, perfluorooctanoic acid; PFOS, perfluorooctane sulfonic acid

We employed WQS models to assess the multiple exposure effects and relative weights of each PFAS compound to metabolic syndrome. In the WQS model for elevated blood pressure, PFHxS was the most influential component, carrying a weight of 0.42, as shown in Fig. 4A. PFOA was another significant contributor with a weight of 0.38. Regarding the model for elevated glucose, two PFAS components had weights exceeding 0.14: PFHxS (weight = 0.73) and PFDA (weight = 0.15) (Fig. 4B). This pattern was similar to the WQS model for reduced HDL cholesterol (Fig. 4C). The weight distribution for elevated triglycerides is outlined in Fig. 4D, where PFHxS and PFNA had the highest weights. This trend was also noticeable in the WQS model for increased waist circumference, as shown in Fig. 4E. These findings elucidate the specific contributions of each PFAS compound to the percentage of WQS for adults with metabolic syndrome.

**Fig. 5:**
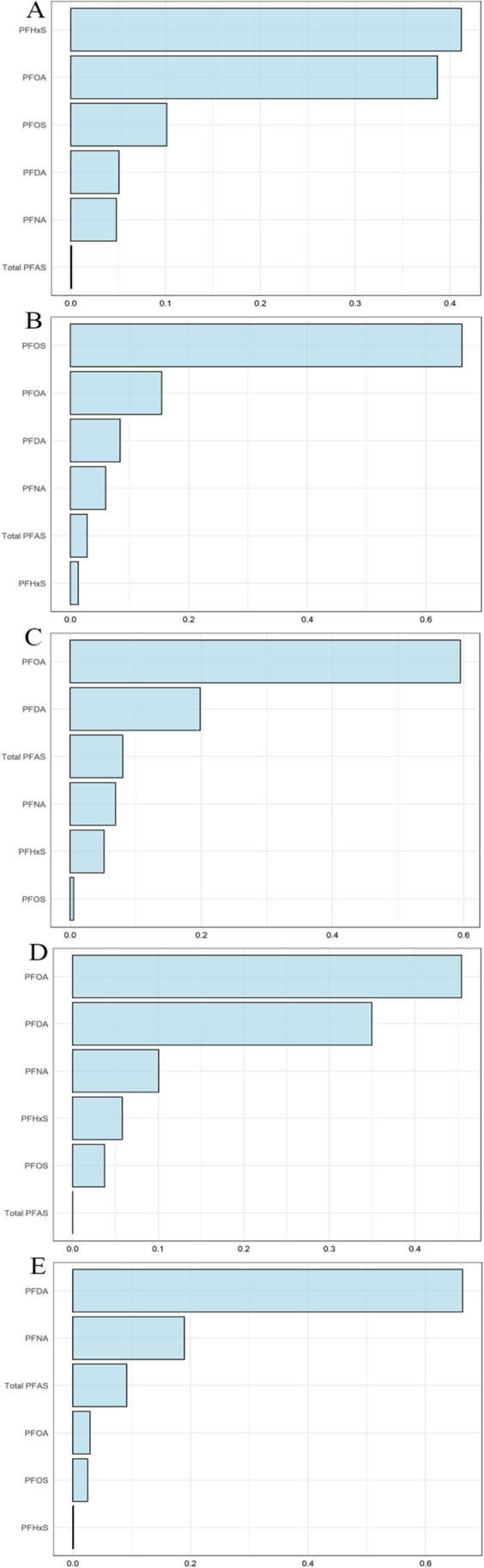
Contribution of PFAS to WQS percentage for metabolic syndrome in adolescent participants. **A**. The WQS index weights for elevated blood pressure. **B**. The WQS index weights for elevated glucose. **C**. The WQS index weights for reduced HDL cholesterol. **D**. The WQS index weights for elevated triglycerides. **E**. The WQS index weights for increased waist circumference. PFAS, per- and polyfluoroalkyl substances; PFDA, perfluorodecanoate; PFHxS, perfluorohexane sulfonate; PFNA, perfluorononanoic acid; PFOA, perfluorooctanoic acid; PFOS, perfluorooctane sulfonic acid

Below is the contribution of each PFAS compound to the percentage of WQS for metabolic syndrome in adolescents. In the WQS model for elevated blood pressure, PFHxS was the most heavily weighted component, with a weight of 0.42 (Fig. 5A). PFOA also had a significant weight, coming in at 0.38. In the model for elevated glucose, both PFOS (weight = 0.66) and PFOA (weight = 0.16) had weights exceeding 0.14 (Fig. 5B). The weight distribution for reduced HDL cholesterol is depicted in Fig. 5C, with PFOA and PFDA holding the top two positions. This pattern was similarly observed in the WQS model for elevated triglycerides, as shown in Fig. 5D. The weight table for increased waist circumference, presented in Fig. 5E, indicated PFDA and PFNA as the top two weighted PFAS components.

### Sensitivity analyses

Overall, sensitivity analyses produced outcomes that were comparable to those of the primary results. In adults, when we treated gender as a categorical variable, the adjusted ORs of MetS associated with PFDA and total PFAS concentrations were [0.63 (0.43, 0.90)] and [0.88 (0.79, 0.98)], respectively (Table S1: Sensitivity analysis i). Using age as a categorical variable in adults, we observed that low levels of PFDA [0.74 (0.58, 0.94)] and high levels of total PFAS [0.72 (0.58, 0.90)] were significantly associated with a reduced prevalence of MetS (Table S1: Sensitivity analysis ii). Negative associations between PFDA and total PFAS exposure and MetS prevalence were also evident when smoking status was treated as a categorical variable; the adjusted ORs were [0.62 (0.42, 0.91)] and [0.84 (0.75, 0.95)] respectively (Table S1: Sensitivity Analysis iii). Lastly, when we considered race as a categorical variable, both PFDA [0.46 (0.29, 0.73)] and total PFAS [0.63 (0.46, 0.88)] were significantly associated with a reduced prevalence of MetS (Table S1: Sensitivity analysis iv).

When age was treated as a categorical variable (Table S2: Sensitivity analysis i), we observed a decrease in the prevalence of MetS in adolescents corresponding to each 1-unit increase in log-transformed levels. This association was evident at high concentration levels of PFDA [0.29 (0.14, 0.58)], PFOA [0.30 (0.10, 0.84)], PFOS [0.20 (0.06, 0.70)], and total PFAS [0.13 (0.03, 0.60)]. Similarly, when race was categorized (Table S2: Sensitivity Analysis ii), we found negative associations between high concentrations of PFDA [0.18 (0.06, 0.57)], PFOA [0.41 (0.17, 0.98)], and total PFAS [0.39 (0.15, 0.98)] and the prevalence of MetS.

## Discussion

In this study, we examined multiple PFASs prevalent in adults and adolescents in the United States and explored their association with MetS and its components. Using consistent results from multiple models, we found that PFDA and total PFAS were associated with a reduced risk of MetS in adults, while PFDA, PFOS, PFOA,and total PFAS were associated with a reduced risk of MetS in adolescents, after controlling for all covariates.

In recent years, researchers have investigated and explored the association between PFAS and MetS. However, the findings have been inconsistent. Some studies have found a positive association between PFAS and MetS (Yang et al. 2018; Chen et al. 2019; Christensen et al. 2019; Yu et al. 2021), but others have shown the opposite association (Lin et al. 2009, Zare Jeddi et al. 2021) or the results did not reach statistical significance (Fisher et al. 2013; Lin et al. 2020; Zare Jeddi et al. 2022). A study from the Chinese C8 Isomer Health Project observed that exposure to PFAS mixtures was positively associated with MetS, especially in women (Yu et al. 2021). Similarly, a small study from the island of Croatia (*n* = 122) found that exposure to PFOS, PFOA, and PFNA was slightly associated with a slightly increased risk of MetS (Chen et al. 2019). Another study based on NHANES (2007–2014) showed that PFNA was associated with an increased risk of MetS even after controlling for multiple PFAS (Christensen et al. 2019). Furthermore, a cross-sectional study by Yang et al. found that elevated PFNA serum levels were linked to a 10.9-fold increase in MetS risk, although this association weakened after age adjustment (Yang et al. 2018). The above studies provide preliminary evidence for a positive association between PFAS and MetS. However, not all studies support the association between PFAS and MetS. A cross-sectional study in Taiwan (*n* = 397) (Lin et al. 2020) and another using the Canadian Health Measures Survey (CHMS) data (Fisher et al. 2013) found no significant links between PFAS and MetS. A 2021 meta-analysis by Maryam et al. (Zare Jeddi et al. 2022) (*n* = 7) similarly concluded that there was no clear association. It's worth noting that while many studies have not found an exact association between PFAS and MetS, they have found varying relationships between PFAS levels and individual components of MetS (Lin et al. 2020, Zare Jeddi et al. 2021). This suggests that PFAS may influence MetS through multiple biological pathways. Overall, the current conclusions regarding the relationship between PFAS and MetS still lack clear uniformity.

Our study explored the association between PFAS and MetS and yielded some surprising results. Specifically, we observed an inverse association between serum PFDA and total PFAS and the risk of developing MetS in adults. This finding provides a new perspective on the relationship between PFAS and MetS. Furthermore, in adolescents, we observed that levels of PFDA, PFOS, PFOA,and total PFAS were also associated with a reduced risk of developing MetS. These findings align with some earlier research. For instance, cross-sectional study from the Veneto region of Italy (Zare Jeddi et al. 2021) (*n* = 876) showed a protective effect of PFOS on the risk of MetS in people aged 20–39 years [0.76 (0.69, 0.85)]. However, this study's limitations include its regional focus and relatively narrow age range, affecting its generalizability. Another cross-sectional study (Lin et al. 2009) based on NHANES data (1999–2000 and 2003–2004) (*n* = 1,443) found that elevated serum PFNA levels were associated with a reduced prevalence of MetS [0.37 (0.21, 0.64)] in adolescents. However, this study relied on older data, potentially affecting its current applicability. In contrast, our study circumvents some of the shortcomings of the aforementioned studies and therefore provides more reliable evidence to support the association between PFAS and MetS risk. Our study addresses some of the limitations seen in these previous works. With a larger sample size and a more diverse participant base, our findings offer more representative and generalizable insights into the relationship between PFAS and MetS. While there are differences between our study and earlier ones, such variations may be attributable to factors like sample characteristics, study design, and data analysis methods. Such studies could offer a more comprehensive understanding of PFAS on human metabolic health and provide new insights for the development of relevant policies and interventions.

Our study revealed distinct associations between various PFAS concentrations and the prevalence of MetS in different age groups. In adults, we observed that low concentrations of PFOS, PFNA, and PFOA, alongside high concentrations of total PFAS and PFHxS, were significantly linked to a reduced prevalence of MetS. Contrastingly, in adolescents, a pronounced association was evident only at elevated concentrations of PFDA, PFOA, PFOS, and total PFAS. Notably, exposure to low and high levels of PFAS had different effects. Previous research has shown varied health effects at different levels of PFAS exposure. For instance, increased serum levels of PFHxS and PFOA have been tied to elevated biomarkers of liver injury (Lin et al. 2010; Gleason et al. 2015; Zhang et al. 2023). Additionally, there's evidence that heightened serum concentrations of PFOA and PFOS are linked with a rising incidence of asthma (Dong et al. 2013; Zhu et al. 2016; Kvalem et al. 2020). Interestingly, many studies have shown that both high and low doses of mixed PFAS exposure have a protective effect on cognitive function (Power et al. 2013; Shrestha et al. 2017; Weng et al. 2022). Furthermore, some studies have found a negative correlation between low-dose PFAS exposure and female infertility (Lum et al. 2017, Tan et al. 2022). The realm of PFAS research is undeniably intricate. PFAS encompasses a multi-substance class where each compound possesses its own chemical structure, potential mechanism of action, and consequent health impacts. Therefore, it's crucial to study individual PFAS compounds in relation to specific diseases to better understand the effects of varying exposure levels on different health outcomes. Despite our increasing knowledge, the underlying mechanisms behind PFAS's health effects largely remain elusive. Our understanding of the relationship between PFAS and MetS is nascent, and any conclusions drawn are based predominantly on our findings in conjunction with previous research, more research is needed to confirm this hypothesis. In our study, we found differing associations between various levels of PFAS and MetS in adults and adolescents. Prior research has similarly explored how the effects of PFAS can vary with age. This discrepancy may arise for several reasons. First, concentrations of certain PFAS may increase with age, given that these chemicals persist in both the environment and the human body. Second, changes in PFAS manufacturing methods since the early 2000s could mean that newer generations are exposed to different types and levels of PFAS compared to older generations.

Our study also explored the association between serum PFAS concentrations and the five components of MetS in both adults and adolescents. In the relevant literature, findings regarding individual metabolic components are inconsistent. However, data from toxicological and epidemiological studies consistently support a hepatic steatosis effect, potentially explaining cholesterol alterations following PFAS exposure (Knutsen et al. 2018; Jain and Ducatman 2019). This association was evident in alterations of total and low-density lipoprotein cholesterol, though the effects on HDL-C and TG remain unclear. In our study, each PFAS demonstrated a negative association with the risk of reduced HDL-C levels in adults. Similarly, among adolescents, PFHxS, PFOS, and total PFAS exhibited this negative trend, consistent with findings from other studies (Lin et al. 2009; Liu et al. 2018; Canova et al. 2020). PFAS molecules have chemical structures similar to fatty acids and may interfere with the fatty acid oxidation pathway (Behr et al. 2020). A common hypothesis suggests that PFAS causes insulin resistance and disorders of lipid metabolism by increasing serum lipocalin levels and oxidative stress (Wang et al. 2017; Stanifer et al. 2018). In addition, previous studies have found that PFOS and PFOA bind to β-lipoprotein, albumin, and liver fatty acid binding protein, which may affect lipid metabolism (Lau et al. 2007). Molecular biology studies have shown that PFOS levels are positively correlated with the cholesterol mobilization transcript NCEH1 and negatively correlated with the cholesterol transport transcript NR1H3 (Fletcher et al. 2013). When it comes to the relationship between PFAS and TG, the results of human studies are inconsistent. Some studies found a positive association between PFAS and TG (Steenland et al. 2009; Zeng et al. 2015), while others did not find a significant relationship (Frisbee et al. 2010). However, a Canadian cross-sectional study showed a negative association between serum PFOS and TG after adjusting for serum levels of n-3 polyunsaturated fatty acids (Château-Degat et al. 2010). In addition, a Swedish long-term follow-up study also found a negative association between PFAS and TG (Donat-Vargas et al. 2019). Similar to the previous studies, our study found that in adolescents, low concentrations of PFDA were associated with a higher risk of elevated triglycerides, but there was no significant association in adults. Population studies focusing on the relationship between PFAS and glycemia are scarce, and most results show no significant association (Lin et al. 2020). Nonetheless, a cross-sectional study of adults based on the C8 Health Cohort (Lind et al. 2014), showed a significant inverse association between higher deciles of PFOA and type 2 diabetes. In vitro studies have shown enhanced differentiation of pancreatic endocrine cells when pancreatic progenitor cells were immersed in oxygen-diffusing membranes containing perfluorocarbons, compared to membranes without perfluorocarbons (Fraker et al. 2007). This is consistent with the reduced risk of elevated serum PFAS and adult EGLU observed in our study.

In recent years, significant progress has been made in toxicological studies on PFAS in wildlife and humans. However, despite the many studies that have been conducted, there is still a knowledge gap regarding the association of specific PFAS with human health. In particular, there are relatively few in vivo and in vitro studies to understand the effects of PFAS on glucose homeostasis. We still do not have a clear understanding of the underlying mechanism of the link between PFAS and glucose homeostasis, but some studies suggest that this link may be partially related to peroxisome activation (Conway et al. 2016). In addition, the hepatotoxicity of PFOS and PFOA is associated with their properties as PPAR-α agonists (Sohlenius et al. 1993; Intrasuksri et al. 1998). PFNA was also found to strongly induce peroxisomal β-oxidation responses in animals (Kudo et al. 2000). However, our findings are not fully consistent with previous studies, suggesting that other or even multiple related pathways may exist between PFAS and MetS. Moreover, our study found differing results between adults and adolescents, which could be attributed to the smaller sample size for adolescents and their different physiological and hormonal profiles. Despite these challenges, further studies are anticipated to deepen our understanding of PFAS's mechanisms of action and its broader implications for human health.

Our study has several strengths that further enhance the reliability and broad applicability of our findings. First, our study is based on data from the NHANES (2003–2018), which represents a multi-ethnic and gender-diverse population in the United States, and thus our findings are more representative of the general population. Second, our study is the first to explore the association between serum PFAS and MetS in adolescents, thereby filling a critical gap in existing research. Finally, we used weighted estimates and adjusted for confounders to overcome bias due to oversampling, thereby enhancing the reliability and precision of our statistical findings. However, some limitations of our study need to be clarified. First, because of the cross-sectional study design, we were unable to determine whether the effect of PFAS on MetS varied over time or to assess its causality. Second, although we adjusted for several confounding factors, we were unable to completely exclude the effects of other confounding factors that could have influenced the results.

## Conclusions

Our study provides new evidence that serum levels of PFAS are associated with a lower prevalence of Metabolic Syndrome (MetS) in a nationally representative sample of U.S. adults and adolescents, after adjusting for confounding factors. In addition, our study showed a significant correlation between PFAS levels and several individual components of MetS, some of which showed protective associations. These results not only corroborate previous research but also yield exciting new insights.

### Supplementary Information

Below is the link to the electronic supplementary material.

Supplementary file1 (DOCX 17 KB)

Supplementary file2 (DOCX 15 KB)

## Data Availability

The dataset supporting the conclusions of this article is available in the NHANES repository, [https://www.cdc.gov/nchs/nhanes].

## References

[CR1] Behr AC, Plinsch C, Braeuning A, Buhrke T (2020). Activation of human nuclear receptors by perfluoroalkylated substances (PFAS). Toxicol Vitro.

[CR2] Canova C, Barbieri G, ZareJeddi M, Gion M, Fabricio A, Daprà F, Russo F, Fletcher T, Pitter G (2020). Associations between perfluoroalkyl substances and lipid profile in a highly exposed young adult population in the Veneto Region. Environ Int.

[CR3] Carrico C, Gennings C, Wheeler DC, Factor-Litvak P (2015). Characterization of Weighted Quantile Sum Regression for Highly Correlated Data in a Risk Analysis Setting. J Agric Biol Environ Stat.

[CR4] Château-Degat ML, Pereg D, Dallaire R, Ayotte P, Dery S, Dewailly E (2010). Effects of perfluorooctanesulfonate exposure on plasma lipid levels in the Inuit population of Nunavik (Northern Quebec). Environ Res.

[CR5] Chen A, Jandarov R, Zhou L, Calafat AM, Zhang G, Urbina EM, Sarac J, Augustin DH, Caric T, Bockor L, Petranovic MZ, Novokmet N, Missoni S, Rudan P, Deka R (2019). Association of perfluoroalkyl substances exposure with cardiometabolic traits in an island population of the eastern Adriatic coast of Croatia. Sci Total Environ.

[CR6] Christensen KY, Raymond M, Meiman J (2019). Perfluoroalkyl substances and metabolic syndrome. Int J Hyg Environ Health.

[CR7] Conway B, Innes KE, Long D (2016). Perfluoroalkyl substances and beta cell deficient diabetes. J Diabetes Complications.

[CR8] Curtin LR, Mohadjer LK, Dohrmann SM, Montaquila JM, Kruszan-Moran D, Mirel LB, Carroll MD, Hirsch R, Schober S, Johnson CL (2012). The National Health and Nutrition Examination Survey: Sample Design, 1999–2006. Vital Health Stat.

[CR9] Donat-Vargas C, Bergdahl IA, Tornevi A, Wennberg M, Sommar J, Koponen J, Kiviranta H, Åkesson A (2019). Associations between repeated measure of plasma perfluoroalkyl substances and cardiometabolic risk factors. Environ Int.

[CR10] Dong GH, Tung KY, Tsai CH, Liu MM, Wang D, Liu W, Jin YH, Hsieh WS, Lee YL, Chen PC (2013). Serum polyfluoroalkyl concentrations, asthma outcomes, and immunological markers in a case-control study of Taiwanese children. Environ Health Perspect.

[CR11] Ervin RB (2009) Prevalence of metabolic syndrome among adults 20 years of age and over, by sex, age, race and ethnicity, and body mass index: United States, 2003–2006. Natl Health Stat Rep (13):1–7 19634296

[CR12] Fändriks L (2017). Roles of the gut in the metabolic syndrome: an overview. J Intern Med.

[CR13] Feng X, Long G, Zeng G, Zhang Q, Song B, Wu KH (2022). Association of increased risk of cardiovascular diseases with higher levels of perfluoroalkylated substances in the serum of adults. Environ Sci Pollut Res Int.

[CR14] Fisher M, Arbuckle TE, Wade M, Haines DA (2013). Do perfluoroalkyl substances affect metabolic function and plasma lipids?–Analysis of the 2007–2009, Canadian Health Measures Survey (CHMS) Cycle 1. Environ Res.

[CR15] Fletcher T, Galloway TS, Melzer D, Holcroft P, Cipelli R, Pilling LC, Mondal D, Luster M, Harries LW (2013). Associations between PFOA, PFOS and changes in the expression of genes involved in cholesterol metabolism in humans. Environ Int.

[CR16] Fraker CA, Alvarez S, Papadopoulos P, Giraldo J, Gu W, Ricordi C, Inverardi L, Domínguez-Bendala J (2007). Enhanced oxygenation promotes beta-cell differentiation in vitro. Stem Cells.

[CR17] Frisbee SJ, Shankar A, Knox SS, Steenland K, Savitz DA, Fletcher T, Ducatman AM (2010). Perfluorooctanoic acid, perfluorooctanesulfonate, and serum lipids in children and adolescents: results from the C8 Health Project. Arch Pediatr Adolesc Med.

[CR18] Ganji V, Zhang X, Shaikh N, Tangpricha V (2011). Serum 25-hydroxyvitamin D concentrations are associated with prevalence of metabolic syndrome and various cardiometabolic risk factors in US children and adolescents based on assay-adjusted serum 25-hydroxyvitamin D data from NHANES 2001–2006. Am J Clin Nutr.

[CR19] Gleason JA, Post GB, Fagliano JA (2015). Associations of perfluorinated chemical serum concentrations and biomarkers of liver function and uric acid in the US population (NHANES), 2007–2010. Environ Res.

[CR20] Glüge J, Scheringer M, Cousins IT, DeWitt JC, Goldenman G, Herzke D, Lohmann R, Ng CA, Trier X, Wang Z (2020). An overview of the uses of per- and polyfluoroalkyl substances (PFAS). Environ Sci Process Impacts.

[CR21] Grundy SM, Cleeman JI, Daniels SR, Donato KA, Eckel RH, Franklin BA, Gordon DJ, Krauss RM, Savage PJ, Smith SC, Spertus JA, Costa F (2005). Diagnosis and management of the metabolic syndrome: an American Heart Association/National Heart, Lung, and Blood Institute Scientific Statement. Circulation.

[CR22] Intrasuksri U, Rangwala SM, O'Brien M, Noonan DJ, Feller DR (1998). Mechanisms of peroxisome proliferation by perfluorooctanoic acid and endogenous fatty acids. Gen Pharmacol.

[CR23] Jain RB, Ducatman A (2019). Dynamics of associations between perfluoroalkyl substances and uric acid across the various stages of glomerular function. Environ Sci Pollut Res Int.

[CR24] Knutsen HK, Alexander J, Barregård L, Bignami M, Brüschweiler B, Ceccatelli S, Cottrill B, Dinovi M, Edler L, Grasl-Kraupp B, Hogstrand C, Hoogenboom LR, Nebbia CS, Oswald IP, Petersen A, Rose M, Roudot AC, Vleminckx C, Vollmer G, Wallace H, Bodin L, Cravedi JP, Halldorsson TI, Haug LS, Johansson N, van Loveren H, Gergelova P, Mackay K, Levorato S, van Manen M, Schwerdtle T (2018). Risk to human health related to the presence of perfluorooctane sulfonic acid and perfluorooctanoic acid in food. Efsa j.

[CR25] Kudo N, Bandai N, Suzuki E, Katakura M, Kawashima Y (2000). Induction by perfluorinated fatty acids with different carbon chain length of peroxisomal beta-oxidation in the liver of rats. Chem Biol Interact.

[CR26] Kvalem HE, Nygaard UC, LødrupCarlsen KC, Carlsen KH, Haug LS, Granum B (2020). Perfluoroalkyl substances, airways infections, allergy and asthma related health outcomes - implications of gender, exposure period and study design. Environ Int.

[CR27] Lau C, Anitole K, Hodes C, Lai D, Pfahles-Hutchens A, Seed J (2007). Perfluoroalkyl acids: a review of monitoring and toxicological findings. Toxicol Sci.

[CR28] Li W, Song F, Wang X, Wang L, Wang D, Yin X, Cao S, Gong Y, Yue W, Yan F, Zhang H, Sheng Z, Wang Z, Lu Z (2018). Prevalence of metabolic syndrome among middle-aged and elderly adults in China: current status and temporal trends. Ann Med.

[CR29] Lin CY, Chen PC, Lin YC, Lin LY (2009). Association among serum perfluoroalkyl chemicals, glucose homeostasis, and metabolic syndrome in adolescents and adults. Diabetes Care.

[CR30] Lin CY, Lin LY, Chiang CK, Wang WJ, Su YN, Hung KY, Chen PC (2010). Investigation of the associations between low-dose serum perfluorinated chemicals and liver enzymes in US adults. Am J Gastroenterol.

[CR31] Lin TW, Chen MK, Lin CC, Chen MH, Tsai MS, Chan DC, Hung KY, Chen PC (2020). Association between exposure to perfluoroalkyl substances and metabolic syndrome and related outcomes among older residents living near a Science Park in Taiwan. Int J Hyg Environ Health.

[CR32] Lind L, Zethelius B, Salihovic S, van Bavel B, Lind PM (2014). Circulating levels of perfluoroalkyl substances and prevalent diabetes in the elderly. Diabetologia.

[CR33] Liu HS, Wen LL, Chu PL, Lin CY (2018). Association among total serum isomers of perfluorinated chemicals, glucose homeostasis, lipid profiles, serum protein and metabolic syndrome in adults: NHANES, 2013–2014. Environ Pollut.

[CR34] Lum KJ, Sundaram R, Barr DB, Louis TA, Buck Louis GM (2017). Perfluoroalkyl Chemicals, Menstrual Cycle Length, and Fecundity: Findings from a Prospective Pregnancy Study. Epidemiology.

[CR35] Power MC, Webster TF, Baccarelli AA, Weisskopf MG (2013). Cross-sectional association between polyfluoroalkyl chemicals and cognitive limitation in the National Health and Nutrition Examination Survey. Neuroepidemiology.

[CR36] Shoeib M, Harner T, Webster GM, Lee SC (2011). Indoor sources of poly- and perfluorinated compounds (PFCS) in Vancouver, Canada: implications for human exposure. Environ Sci Technol.

[CR37] Shrestha S, Bloom MS, Yucel R, Seegal RF, Rej R, McCaffrey RJ, Wu Q, Kannan K, Fitzgerald EF (2017). Perfluoroalkyl substances, thyroid hormones, and neuropsychological status in older adults. Int J Hyg Environ Health.

[CR38] Sohlenius AK, Eriksson AM, Högström C, Kimland M, DePierre JW (1993). Perfluorooctane sulfonic acid is a potent inducer of peroxisomal fatty acid beta-oxidation and other activities known to be affected by peroxisome proliferators in mouse liver. Pharmacol Toxicol.

[CR39] Stanifer JW, Stapleton HM, Souma T, Wittmer A, Zhao X, Boulware LE (2018). Perfluorinated Chemicals as Emerging Environmental Threats to Kidney Health: A Scoping Review. Clin J Am Soc Nephrol.

[CR40] Steenland K, Tinker S, Frisbee S, Ducatman A, Vaccarino V (2009). Association of perfluorooctanoic acid and perfluorooctane sulfonate with serum lipids among adults living near a chemical plant. Am J Epidemiol.

[CR41] Tan Y, Zeng Z, Liang H, Weng X, Yao H, Fu Y, Li Y, Chen J, Wei X, Jing C (2022). Association between perfluoroalkyl and polyfluoroalkyl substances and women’s infertility, NHANES 2013–2016. Int J Environ Res Public Health.

[CR42] Wang X, Liu L, Zhang W, Zhang J, Du X, Huang Q, Tian M, Shen H (2017). Serum metabolome biomarkers associate low-level environmental perfluorinated compound exposure with oxidative /nitrosative stress in humans. Environ Pollut.

[CR43] Weng X, Liang H, Tan Y, Chen J, Fei Q, Liu S, Guo X, Wen L, Wu Y, Jing C (2022). Mixed effects of perfluoroalkyl and polyfluoroalkyl substances exposure on cognitive function among people over 60 years old from NHANES. Environ Sci Pollut Res Int.

[CR44] Yan S, Zhang H, Zheng F, Sheng N, Guo X, Dai J (2015). Perfluorooctanoic acid exposure for 28 days affects glucose homeostasis and induces insulin hypersensitivity in mice. Sci Rep.

[CR45] Yang Q, Guo X, Sun P, Chen Y, Zhang W, Gao A (2018). Association of serum levels of perfluoroalkyl substances (PFASs) with the metabolic syndrome (MetS) in Chinese male adults: A cross-sectional study. Sci Total Environ.

[CR46] Yu S, Feng WR, Liang ZM, Zeng XY, Bloom MS, Hu GC, Zhou Y, Ou YQ, Chu C, Li QQ, Yu Y, Zeng XW, Dong GH (2021). Perfluorooctane sulfonate alternatives and metabolic syndrome in adults: New evidence from the Isomers of C8 Health Project in China. Environ Pollut.

[CR47] Zare Jeddi M, Dalla Zuanna T, Barbieri G, Fabricio ASC, Daprà F, Fletcher T, Russo F, Pitter G, Canova C (2021). Associations of perfluoroalkyl substances with prevalence of metabolic syndrome in highly exposed young adult community residents-a cross-sectional study in Veneto Region, Italy. Int J Environ Res Public Health.

[CR48] Zare Jeddi M, Soltanmohammadi R, Barbieri G, Fabricio ASC, Pitter G, DallaZuanna T, Canova C (2022). To which extent are per-and poly-fluorinated substances associated to metabolic syndrome?. Rev Environ Health.

[CR49] Zeng XW, Qian Z, Emo B, Vaughn M, Bao J, Qin XD, Zhu Y, Li J, Lee YL, Dong GH (2015). Association of polyfluoroalkyl chemical exposure with serum lipids in children. Sci Total Environ.

[CR50] Zeng G, Zhang Q, Wang X, Wu KH (2022). The relationship between multiple perfluoroalkyl substances and cardiorespiratory fitness in male adolescents. Environ Sci Pollut Res Int.

[CR51] Zhang L, Ren XM, Wan B, Guo LH (2014). Structure-dependent binding and activation of perfluorinated compounds on human peroxisome proliferator-activated receptor γ. Toxicol Appl Pharmacol.

[CR52] Zhang X, Zhao L, Ducatman A, Deng C, von Stackelberg KE, Danford CJ, Zhang X (2023). Association of per- and polyfluoroalkyl substance exposure with fatty liver disease risk in US adults. JHEP Rep.

[CR53] Zhu Y, Qin XD, Zeng XW, Paul G, Morawska L, Su MW, Tsai CH, Wang SQ, Lee YL, Dong GH (2016). Associations of serum perfluoroalkyl acid levels with T-helper cell-specific cytokines in children: By gender and asthma status. Sci Total Environ.

