# Supplementary material for: Association of per- and polyfluoroalkyl substance exposure with metabolic syndrome and its components in adults and adolescents
Source: Environ Sci Pollut Res Int. 2023 Oct 16;30(52):112943–58. doi: 10.1007/s11356-023-30317-x (PMC10643431; doi:10.1007/s11356-023-30317-x)
Supplement: Supplementary file 1 — Supplementary file1 (DOCX 17 KB) [file 11356_2023_30317_MOESM1_ESM.docx]

**Table S1**. Associations between PFAS exposures and risk of [metabolic syndrome](javascript:;) in adult participants ([sensitivity analysis](javascript:;) i-iv).

|  | Ln-transformed  OR (95%CI) | Tertile 1 | Tertile 2  OR (95%CI) | Tertile 3  OR (95%CI) | *P* for tend |
| --- | --- | --- | --- | --- | --- |
| Sensitivity analysis i | |  |  |  |  |
| PFDA | **0.63 (0.43, 0.90)** | 1.00 (Reference) | **0.74 (0.58, 0.93)** | 0.82 (0.67, 1.01) | 0.1992 |
| PFHxS | 0.87 (0.73, 1.02) | 1.00 (Reference) | 0.81 (0.64, 1.03) | 0.80 (0.64, 1.01) | 0.1849 |
| PFNA | 0.92 (0.74, 1.15) | 1.00 (Reference) | **0.77 (0.62, 0.96)** | 0.88 (0.71, 1.09) | 0.5771 |
| PFOA | 0.92 (0.79, 1.09) | 1.00 (Reference) | **0.79 (0.63, 0.99)** | 0.91 (0.73, 1.13) | 0.4251 |
| PFOS | **0.90 (0.81, 0.99)** | 1.00 (Reference) | 0.84 (0.68, 1.05) | 0.80 (0.64, 1.00) | 0.0538 |
| Total PFAS | **0.88 (0.79, 0.98)** | 1.00 (Reference) | **0.76 (0.61, 0.95)** | 0.87 (0.67, 1.12) | **0.0051** |
| Sensitivity analysis ii | |  |  |  |  |
| PFDA | **0.51 (0.31, 0.82)** | 1.00 (Reference) | **0.74 (0.58, 0.94)** | 0.77 (0.58, 1.01) | **0.0257** |
| PFHxS | 0.99 (0.80, 1.22) | 1.00 (Reference) | 0.88 (0.69, 1.13) | 0.91 (0.69, 1.20) | 0.5248 |
| PFNA | 0.82 (0.62, 1.08) | 1.00 (Reference) | **0.71 (0.55, 0.91)** | 0.91 (0.70, 1.18) | 0.5128 |
| PFOA | 0.87 (0.72, 1.06) | 1.00 (Reference) | **0.71 (0.55, 0.92)** | 0.92 (0.71, 1.18) | 0.5038 |
| PFOS | 0.92 (0.82, 1.04) | 1.00 (Reference) | 0.87 (0.68, 1.11) | 0.84 (0.65, 1.09) | 0.1736 |
| Total PFAS | 0.90 (0.79, 1.03) | 1.00 (Reference) | 0.94 (0.74, 1.19) | **0.72 (0.58, 0.90)** | 0.2803 |
| Sensitivity analysis iii | |  |  |  |  |
| PFDA | **0.62 (0.42, 0.91)** | 1.00 (Reference) | **0.78 (0.62, 0.99)** | 0.95 (0.77, 1.16) | **0.0319** |
| PFHxS | 1.01 (0.85, 1.20) | 1.00 (Reference) | 0.91 (0.74, 1.13) | **0.76 (0.60, 0.95)** | 0.0503 |
| PFNA | 1.02 (0.81, 1.29) | 1.00 (Reference) | **0.66 (0.53, 0.83)** | 1.08 (0.87, 1.34) | 0.0861 |
| PFOA | 1.02 (0.87, 1.21) | 1.00 (Reference) | **0.68 (0.55, 0.86)** | 1.08 (0.87, 1.34) | 0.0895 |
| PFOS | 0.99 (0.89, 1.10) | 1.00 (Reference) | 0.92 (0.74, 1.14) | 0.97 (0.78, 1.20) | 0.0600 |
| Total PFAS | **0.84 (0.75, 0.95)** | 1.00 (Reference) | 0.86 (0.69, 1.07) | **0.70 (0.56, 0.88)** | **0.0020** |
| Sensitivity analysis iv | |  |  |  |  |
| PFDA | **0.46 (0.29, 0.73)** | 1.00 (Reference) | **0.75 (0.60, 0.93)** | 0.84 (0.66, 1.06) | **0.0122** |
| PFHxS | 0.88 (0.74, 1.05) | 1.00 (Reference) | 0.80 (0.63, 1.02) | 0.80 (0.63, 1.01) | 0.1084 |
| PFNA | 0.92 (0.73, 1.18) | 1.00 (Reference) | **0.73 (0.57, 0.93)** | 0.94 (0.74, 1.18) | 0.8483 |
| PFOA | 0.95 (0.79, 1.13) | 1.00 (Reference) | **0.76 (0.59, 0.96)** | 0.96 (0.76, 1.22) | 0.7857 |
| PFOS | 0.92 (0.82, 1.02) | 1.00 (Reference) | **0.76 (0.60, 0.96)** | 0.83 (0.66, 1.05) | 0.1713 |
| Total PFAS | **0.63 (0.46, 0.88)** | 1.00 (Reference) | 0.80 (0.63, 1.01) | **0.74 (0.58, 0.93)** | 0.0819 |

PFAS, per- and polyfluoroalkyl substances; PFDA, perfluorodecanoate; PFHxS, perfluorohexane sulfonate; PFNA, perfluorononanoic acid; PFOA, perfluorooctanoic acid; PFOS, perfluorooctane sulfonic acid.
