# Supplementary material for: Association of per- and polyfluoroalkyl substance exposure with metabolic syndrome and its components in adults and adolescents
Source: Environ Sci Pollut Res Int. 2023 Oct 16;30(52):112943–58. doi: 10.1007/s11356-023-30317-x (PMC10643431; doi:10.1007/s11356-023-30317-x)
Supplement: Supplementary file 2 — Supplementary file2 (DOCX 15 KB) [file 11356_2023_30317_MOESM2_ESM.docx]

**Table S2**. Associations between PFAS exposures and risk of [metabolic syndrome](javascript:;) in adolescent participants ([sensitivity analysis](javascript:;) i-ii).

|  | Ln-transformed  OR (95%CI) | Tertile 1 | Tertile 2  OR (95%CI) | Tertile 3  OR (95%CI) | *P* for tend |
| --- | --- | --- | --- | --- | --- |
| Sensitivity analysis i | |  |  |  |  |
| PFDA | 0.64 (0.10, 3.92) | 1.00 (Reference) | 0.52 (0.06, 4.46) | **0.29 (0.14, 0.58)** | **0.0005** |
| PFHxS | 1.09 (0.75, 1.59) | 1.00 (Reference) | 0.57 (0.27, 1.20) | 0.57 (0.27, 1.19) | 0.4122 |
| PFNA | 1.45 (0.70, 3.01) | 1.00 (Reference) | 0.88 (0.40, 1.90) | 0.65 (0.28, 1.52) | 0.3119 |
| PFOA | 0.99 (0.61, 1.62) | 1.00 (Reference) | 0.67 (0.32, 1.39) | **0.30 (0.10, 0.84)** | 0.6664 |
| PFOS | 0.93 (0.70, 1.25) | 1.00 (Reference) | 0.96 (0.47, 1.95) | **0.20 (0.06, 0.70)** | 0.5349 |
| Total PFAS | 0.98 (0.71, 1.36) | 1.00 (Reference) | 0.94 (0.47, 1.90) | **0.13 (0.03, 0.60)** | 0.7110 |
| Sensitivity analysis ii | |  |  |  |  |
| PFDA | 0.02 (0.00, 258.56) | 1.00 (Reference) | 1.09 (0.11, 10.46) | **0.18 (0.06, 0.57)** | 0.1422 |
| PFHxS | 0.59 (0.05, 7.33) | 1.00 (Reference) | 0.59 (0.24, 1.48) | 0.45 (0.19, 1.08) | 0.0588 |
| PFNA | 0.05 (0.00, 7.20) | 1.00 (Reference) | 0.70 (0.26, 1.86) | 1.04 (0.45, 2.44) | 0.7668 |
| PFOA | 0.05 (0.00, 1.28) | 1.00 (Reference) | 0.73 (0.29, 1.81) | **0.41 (0.17, 0.98)** | **0.0450** |
| PFOS | 1.95 (0.51, 7.49) | 1.00 (Reference) | 0.93 (0.39, 2.22) | 0.47 (0.19, 1.15) | 0.1083 |
| Total PFAS | 1.42 (0.29, 6.94) | 1.00 (Reference) | 0.79 (0.32, 1.93) | **0.39 (0.15, 0.98)** | 0.0768 |

PFAS, per- and polyfluoroalkyl substances; PFDA, perfluorodecanoate; PFHxS, perfluorohexane sulfonate; PFNA, perfluorononanoic acid; PFOA, perfluorooctanoic acid; PFOS, perfluorooctane sulfonic acid.
